# Supplementary material for: Natural emotion vocabularies as windows on distress and well-being
Source: Nat Commun. 2020 Sep 10;11:4525. doi: 10.1038/s41467-020-18349-0 (PMC7483527; doi:10.1038/s41467-020-18349-0)
Supplement: Supplementary file 1 — Supplementary Information [file 41467_2020_18349_MOESM1_ESM.pdf]

Supplementary Information for:

Natural Emotion Vocabularies as Windows on Distress and Well-being

By Vine *et al.*

Supplementary Table 1. Words and Word Stems for Emotion Vocabulary (EV) Computations

Vocabulate Dictionary Item	Neg	Pos	Anx\ Fear	Anger	Sadness	Neg Undiff
afraid	X		X			
aggravat*	X			X		
agitat*	X					
agony	X					
alarm*	X		X			
alone	X				X	
anger|angr*	X			X		
anguish*	X				X	
annoy*	X			X		
anxi*	X		X			
apath*	X				X	
appall*	X		X	X		
apprehens*	X		X			
asham*	X					
ashame*	X					
aversi*	X					
bitter	X				X	
contempt	X			X		
crushed	X				X	
depress*	X				X	
despair	X				X	
desperate*	X					
despis*	X			X		
devastat*	X				X	
disappoint*	X				X	
discourage*	X				X	
disgust	X					
dishearten*	X				X	
disillusion*	X				X	
dislike|disliked|dislikes|disliking	X					
dismay*	X				X	
dissatisf*	X				X	
distraught	X					
disturb*	X					
doom	X		X		X	
dread*	X		X			
embarrass*	X					
envy|envie*|envious	X					
fear|feared|fearful|fearing|fears	X		X			
frantic	X		X			
fright	X		X			
frustrat*	X			X		
fury|furious	X			X		
gloom	X				X	
grief|griev*	X				X	
guilt*	X					

hate|hated|hateful|hater|hates|hating|hatred	X			X		
heartbreak*|heartbroke*	X				X	
helpless	X				X	
homesick	X				X	
hopeless	X				X	
horror*|horrif*	X		X			
hostil*	X			X		
humiliat*	X					
hurt	X					
insecur*	X					
intimidat*	X					
irrita*	X			X		
jealous*	X					
lone*	X				X	
longing	X				X	
loss	X				X	
mad|maddening|madder|maddest	X			X		
melanchol*	X				X	
miss	X				X	
mourn	X				X	
nervous	X		X			
offend|offence|offens*	X			X		
outrag*	X			X		
panic	X		X			
paranoi*	X		X			
petrif*	X		X			
phobi*	X		X			
pity	X					
rage	X			X		
regret*	X				X	
reluctan*	X					
remorse*	X				X	
resent*	X			X		
restless	X					
sad|sadde*|sadly|sadness	X				X	
scared|scare|scaring|scary	X		X			
shock*	X					
solemn*	X				X	
sorrow	X				X	
startl*	X		X			
stunned	X					
terror|terrifies|terrified|terrify*	X		X			
turmoil	X					
unhapp*	X				X	
unlov*	X				X	
woe*	X				X	
worr*	X		X			
yearn*	X				X	
awful						X
bad						X
crap|crappy*						X

craz*						X
distress*						X
emotional						X
horribl*						X
numb|numbed|numbs|numbing|numbly|numbness*						X
overwhelm*						X
rotten						X
stress*						X
sucky						X
terribl*						X
unpleasant						X
upset*						X
admir*		X				
ador*		X				
affection*		X				
amor*		X				
amus*		X				
appreciat*		X				
cheer|cheery|cheeriness*|cheerily|cheerful*		X				
cherish*		X				
compassion*		X				
confident*|confidence		X				
content|contented*|contentment		X				
curious*		X				
delight*		X				
eager*		X				
ecsta*		X				
elated*|elation		X				
enjoy*		X				
enraptur*		X				
entertain*		X				
enthrall*		X				
enthus*		X				
euphori*		X				
excit*		X				
exhil*		X				
fond|fondly|fondness*		X				
glad|gladly|gladness*		X				
glee|gleeful*		X				
gratef*|gratitude*		X				
happy|happi*		X				
hope|hopeful*|hopefulness		X				
inspired*		X				
interest*		X				
joll*		X				
joy*		X				
keen*		X				
love|loved|loves|loving*		X				
merr*		X				
optimis*		X				
passion*		X				

peace*		X				
pleased|pleasant*|pleasur*		X				
proud*|pride*		X				
relief|reliev*		X				
resolv*		X				
satisf*		X				
secur*		X				
serene|serenit*		X				
surpris*		X				
thankf*		X				
thrill*		X				
tranquil*		X				
triumph*		X				
vigor|revigor*|invigor*|vigour		X				

Note. Word stems are partial words terminated by an asterisk (*). Use of an asterisk at the end of the word or word stem signals our software program to ignore all subsequent letters. For example, “thankf*” will capture uses of “thankful,” “thankfully,” and “thankfulness,” but not “thanks” or “thank you.” Variants separated by the symbol “|” were counted as instances of the same emotion word, where an asterisk would have provided insufficient specificity.

Supplementary Methods

Excerpted Writing Samples from Study 1 (Stream-of-Consciousness Essays)

Potentially identifying details are redacted using square brackets to protect writer confidentiality. Participant's negative and positive EV scores and corresponding percentile ranks are indicated to aid interpretation.

Writing Sample 1 (Negative EV = 1.13, 95th percentile; Positive EV = 0.57, 63rd percentile)
My feelings right now are both really sad and excited. Sad because a guy I liked asked me to be his date to something, but I can't go because I already have plans to go to a music festival that weekend. Now I am worried because he will probably ask someone else and I missed my chance. I seem to worry a lot about not being good enough like right now. Or worry about people really liking me. I feel like there's usually someone cooler out there than me. But I am also excited because I am going to [swimming pool name] for the first time today with my best friend and some other friends. I'm hoping it'll get my mind off my disappointment. I love the outdoors and can't wait to hammock. Something else that has been bothering me lately is my desire to start a blog and be on a magazine. I love photography and want to do both of those things to further advance my photography career. But I'm scared to try out for a magazine because I'm worried I won't be good enough (yet again). I am also concerned about my blog. I feel like I only get bursts of motivation and don't really stick with anything. One of my fears is that I won't work hard enough to get where I want to be and be who I dream of being. Which seems like a silly fear at times because all I have to do to change that is work hard. But I'm afraid I won't have the passion or I'll lose the passion to work hard. But I want to be a go-getter and hope that I will work hard and stick with my blog so that I can one day be a [magazine name] blogger and photographer and maybe even be a photographer for [other magazine]. Now back to my sadness about the guy asking me out because that's what is predominately on my mind at the moment. The one guy I actually like and is really cool actually asks me to something and I can't go. I may not get this chance again and he will probably ask another girl and they will end up liking each other. I also feel a sense of guilt because a guy back home asked me to be his girlfriend, but I just wanted to keep it unofficial because we are far away from each other and I've never been in a relationship. But now I'm falling for guys here and feel bad. I like the guy back home and feel like I should go out with him because I caused drama in my friend group by liking him (my best friend thought I was mad at her for hanging out with him, but I'm just insecure. And he was actually my friend's prom date, but they ended up not going out after prom), but sadly I feel like I may be almost losing interest already. This happens a lot. I'm afraid of mundanity and this is an example of that because I feel like I'm already getting bored. This is why I'm afraid I'll never commit to anything or anyone. It seems like I lose passion and drive fast. All I want to do is find my passion in life and want to work hard and learn and stick with it. I'm hoping everything will work out with the boy situation. I really hope the guy here asks me to something else and maybe even asks me to the event I can't go to this year, next year. And if I do find someone else here that I will be able to tell the guy back home and we will still be good friends and it won't mess up the friend group. I hate confrontation so I feel like if I wanted to end things with him I wouldn't be able to or I would handle it badly. I'm already an emotional and insecure person and college is just a huge emotional rollercoaster of loneliness, missing family and friends. But also excitement of meeting new people and living in a new and adventurous city. I guess I'll just hope for the best and hope

that I'll truly have the peace of mind to believe that everything will work out how it's meant to be. Hopefully I will strengthen my relationship with God and have the faith and peace to trust Him and pray to him, letting life happen as it may. I'm scared of letting people in because they will decide they don't like the real me. I'm scared of hanging out one on one with people, especially guys, because I'm scared of awkward situations. I'm scared when people seem to really like me because I'm afraid I won't be able to deliver and won't meet their expectations. I hate feeling like this all the time and pray that God will give me peace. I just wish I was more of a free spirit and just let things happen and not worry. Be a free spirit and hang out with anyone and everyone, never being afraid or living in the "confines of fear" like Ben Howard says in his song. I don't want to live a mundane life or live in the "confines of fear," never striving to reach my full potential or being to afraid to hang out with certain people because of the possibility of "awkwardness." Well I just released all my overall worries, fears and insecurities and new occurrences that are bothering me that I usually unleash on my two best friends or my mom. I'm hoping this little venting session helped my worries even though I am about to talk to one of my best friends about it and it might make me upset again. But she usually has the best advice so she will help me. I miss her often... she lives in [location] and I haven't talked to her since I got to college really because we have both been so busy. I don't know what's going on in her life which is weird because we usually talk every day. But we will talk now and then I'm busy the rest of the day and will be really stressed with all my homework and readings I have to do tomorrow.

Writing Sample 2 (Negative EV = 0.72, 73rd percentile; Positive EV = 0.48, 52nd percentile)
I don't know really how I feel. College life to me is just another thing I have to do. I really can't wait for when I don't have to study anymore and I get to work a job. I love working for some reason. For like the last year and a half, I have been a busser/server for [restaurant]. Now it was only a part-time job but I couldn't wait till the end of the week when I would get to work. Most people would be mad that they had to work on the weekends. The only days [restaurant] was open was thursday, friday, saturday, and some sundays. I absoutely enjoyed working. I lost most of my weekend to work and to some crazy school assignment if i had one. I might be alittle over exaggerating the crazy part but I almost never did a assignment until the very very end. I would somehow find myself finishing the assignment on the day it was due. I stopped finishing it the night before. That was just too early for me. But when it comes to working I go above and beyond for everyone. Starting from my first season at [restaurant], I was always the one that would stay after and make my small group of friends that had the job stay with me if our manager ever needed something done. I tried to work everyday that I could. Somehow every season I found myself missing a couple big days because of school activities and stuff which made me feel bad. Whenever i had to ask for a day off, it just made me want to work even harder. Even though i would miss a day for something like the SAT test or a theatre competition. After my first season, my manager told me and my group of about 3 others that she would promote us to server when we become 18. My first season I barely turned 17. So, i was a busser for 2 more seasons. But I worked so hard that my manager said we were probably her hardest working employees ever. I really can't wait to work. I know its kinda bad to dislike class and learning when it really is going to be the reason why I will do better in my profession but I just want to teleport myself to the end of college. I dont know why. I know college is gonna be extremely fun and the people i meet here will probably some of my long time friends when i get old but I'm already ready to start working. Hmmmm.. I guess College is going alright for me. I'm normally a very loud, talkative guy but I have these modes where I'm super silent. I've been in

this super silent since I got here. Also I have this weird nervous thing. When i'm acting in a show and stuff I am NEVER nervous. NEVER! Alot of people know me as never being shy but man i have some moments where I get so nervous I can barely control it. I went to a business camp [camp name] and the team i was on had to give a presentation of our plan to get kids to stop smoking. I got the task of being the introduction. I am that type of guy. The guy that would get you hype for something. I planned it out perfectly. When I started my body started shaking crazy! The only reason why no one saw it was because I was moving around alot in my intro. When I finished, it took me a second to relax. I'm normally a really really calm guy. No one can get me rattled but there are certain situations where out of no where i get really really nervous. The intro was great though. Everyone loved it and they were surprised at how good it was. You would think that the reaction I got would calm my nerves the next time but nope. I dont remember all the times when i got nervous like that but it definitely happened. In times where I should be nervous, I'm not. In times where I shouldn't be nervous, I'm nervous as heck. I know I am a really special (special as in different) kind of person. I almost never get mad. I stay happy pretty much 24/7. Most of the people I know have nerver seen me get mad or sad. I have like multiple modes. I wouldnt call them personalities because I'm the same person in each mode. I'm either quiet or loud. I need to get out of this quiet mode here though cause there are alot of people i need to meet and become friends with. People are leaving openings for me to become friends and stuff but i dont do anything about it and i'm probably not leaving any opening for them. Anyways its been way passed 20 min so imma stop.

Writing Sample 3 (Negative EV = 0.56, 57th percentile; Positive EV = 0.74, 81st percentile)
I wanted to go to bed early tonight! Well, at least I still have time to finish the assignment before 11:59pm. As much as I wanted to take this class, it is a bit disheartening that I don't have an A in it right now. It's going to be difficult to bring up my grade, especially since I'm going to [foreign country] next month for two weeks. I wonder if I'll be able to attend the live class and do the benchmarks while I'm in [country]...I hope so, but it's really unlikely. I'm going to be pretty disappointed if I don't get an A in this course, but oh well, I suppose grades aren't the most important thing. At least I'm learning, and I'm definitely going to pass the class, so I'll be able to take more psychology courses in the spring and next fall. Hmm, what else is on my mind? Well, now that I mentioned my trip to [country] I'm getting pretty excited, even though I'm very tired from today. I guess I'll think about being excited for my trip and then about my day today. (Or does that ruin this exercise because I've planned out what I'd write about? Well, it's not my fault, these are my thoughts, perceptions, and feelings at the moment...) [Country] is going to be awesome. [Name] is turning 100 years old and so much of the [last name] Family is getting together to celebrate in [city, country], and then going to [another location]. I suppose whoever reads this will have no idea why I'm so excited for this because they probably have no idea what [name] is. Well, they'll at least be able to understand the excitement surrounding a 100 year anniversary. I wonder if I don't explain what [name] is, if the reader will be curious enough to look it up after he or she finishes reading all of these \"stream of consciousness.\" Look it up! It's what I'm dedicating my life to! [Redacted content.] Family and friends are truly the most important thing in life. The joy to have found out what I was made to do with my life, what God created me to do, coupled with the support and love of my family and friends, is indescribable. Every day the joy and love I feel in my heart grows more and more. I have so much to be thankful for. (Can you end a sentence with a preposition or not? I'm going to do.) What else? What is this assignment even for? I wonder what it measures. What's going to happen if I don't

click submit to instructor before the time is up? I hope it submits it without clicking that button like it does with the benchmarks. Hmm, what more is there to say? Or I suppose think, perceive for feel? Well, I said I would talk about the day as well. Today was great. It was great to have lunch with a friend, and I dropped a course which frees up my schedule a bit more to do [redacted] things. I felt particularly spiritual today, which is always great for someone who's made the decision I have. I suppose that's actually great for anyone if that's what they're looking for. (Whoops, I ended another sentence with a preposition again.) I wonder what my brothers are doing...I hope they're doing good and are happy. It's tough to see them often even though they also go to [university name], but they're doing their own thing. And we do see each other at least once a week. I hope they're happy. And I hope my parents are happy today as well. Come to think about it, I hope all my family and friends are happy. Hm, and what about those who are afraid and alone? What's my role? I suppose I can pray for those who have no one to pray for them, but is there anything else God is calling me to do for them? I have to always keep my hand on the pulse of the times and my ear to God's heart. Ah, I'm tired. Is this almost over?

Writing Sample 4 (Negative EV = 0.72, 73rd percentile; Positive EV = 0.10, 6th percentile)
My leg started to fall asleep just now and so i moved it and im thinking about that weird almost cold feeling that happens when blood returns to the part of the body that was asleep before. Im in a library and im self-conscious about my sniffling because i've had a cold for a month and a half and some days it is worse than others and today i have a sniffle. Last night was also stressful because my best friend was really drunk and making a bad decision and we, my friend and I, tried to tell her it was a bad idea but instead of us helping we made it a lot worse and she left and cried. But we've talked today and she said she was fine and I trust her because we're really honest with each other so I know she wouldn't bullshit me. The drama with the other guy was also a big deal last night because i finally got to talk to him about it, and now I think we're okay and we can be normal, but I'm not sure because I texted him late last night and he never responded but then again he's pretty bad at responding in general. And it sucks because he is starting pledgeship with a fraternity so i'll probably never see him which makes it a lot harder to see how we actually feel about eachother.. but i really think we're fine and normal. He makes me nervous because he isn't that strong of a person and he's very easily stressed so I am scared to see the effects of college on him. One of my new pledge-sisters just walked into this library and i said hi and thats what im thinking now. Im also a little worried because i've been listening to the same playlist on spotify for awhile now and i hate it when i get sick of music. I don't want to over do myself on these songs, but it's so good that i just want to keep listening. A song just came on that reminds me of one of my friends that goes to [university name]. He's great. I stopped in [City] on my way back to [City] the other day and I saw him, and it was fun but a little awkward cause we didnt have that much to talk about. Im slightly annoyed now because typing on this new laptop is hard and i keep messing up. the keys are smaller than my macbook's keys. Wooohoo almost done only 10 more minutes. Ha. Now im watching these guys at the table in front of me. they look like they're in a study group or something but they dont really look like they're getting much done.. the guy across the table looks like hes working pretty hard. good for him. studious ha. last night i wore dreads to a party and i braided my whole head and it looked ridiculous and I'm a little bummed cause I almost left it in for class and then last minute late last night i decided against it. But i was wearing the dreads when i went and talked to my guy friend about all that drama and i could barely take myself seriously. And it was raining so hard last night that when i walked into his apartment I wasn't wearing shoes (didnt want to ruin them) and i was soaked to the bone. Soo

wet it was humorous. Ugh the amount of times i have been barefoot in [building name] is unreal. its nasty i dont know how that keeps happening. I have this crick in my neck and it hurts when im just sitting here. I blame psychology for my neck crick. just kidding but it does hurt.

Samples of Emotion Words Captured from Individual Participants in Studies 1 (Stream-of-Consciousness Essays) and 2 (Blogs)

**Study 1 Captured Word Samples**			
**Total Word Count**	**Negative EV Score** Sample mean .55 (*SD* = 0.36, range = 0 to 5.71)	**Positive EV Score** Sample mean .52 (*SD* = 0.34, range = 0 to 3.75)	**Emotion Words Captured**
890	0.00	0.77	*happy excited thankful overwhelming love unpleasant loves hope loving love love love love hope hope hope happiness love joy happiness happy love love love love love love*
1101	0.33	0.33	*stressed nervous stressing stressing stresses stressed miss terrible miss miss miss loving stresses miss bad interesting stress stressed bad hate miss miss love stressing miss love stresses miss stressed happy*
863	0.40	0.27	*hope love love love miss hate miss miss miss love love love love love love bad bad scared scared loved love love hope*
449	1.46	0.73	*stressed angry happy hopeful lonely angrier frustrated anxiety love lonely love love love alone love worry worry worry*
469	1.57	0.45	*glad sad mourn loss stressed guilty nervous worries worried guilty love stressed helpless bad*
225	1.94	0.49	*anxious homesick worried miss miss glad*
855	5.71	2.86	*stress sad depressed happy sad anxious hopeless restless worried satisfied annoyed paranoia happiness joy peace*

**Study 2 Captured Word Samples**			
**Total Word Count**	**Negative EV Score** Sample mean .29 (*SD* = 0.21, range = 0 to 2.66)	**Positive EV Score** Sample mean .33 (*SD* = 0.21, range = 0 to 2.49)	**Emotion Words Captured**
10,599	0.15	0.14	*peaceful peaceful alone sadly sad proudly love interested surprise interest hated bad miss sad love love hurt afraid hurt hurt crap scared hurt afraid hope scary love love love love love love love love love interesting hate annoying annoying crazy bad bad awful bad terribly bad sad annoying miss lonely excite miss pity depressed miss sad adore hate joy delight pleasures sad sad adore sad crazy bad sad love love love loved hurt adorable loves sad thrill love guilty scare bad glad miss bad crazy crazy crazy excited hurt crazy bad bad crazy crazy miss bad enjoying stressed peace love happy scary agony hope sad crazy irritated love surprise bad bad happy loves mad bad crazy grief happy loves love love crazy interesting love crazy love love love love excited*

			loved happy love bad love joyous bad enjoyed
1990	0.15	0.35	*doom love shocked shock bad happy loved security crazy crap surprise love bad crazy love sad sadder enjoying love enjoy enjoy hope interesting*
663	0.15	0.45	*hope surprised relieved disappointed*
646	1.39	0.46	*crap love worrying crazy crap agony melancholy lonely melancholy sadness happiness crap happier angry crap crap irritating sad hate irritating miss glad*
682	1.76	0.44	*fear anxiety fears overwhelming distressing shock numbness loss sadness shock numbness grieve loved loss anger fear loss anger loved anxiety anger fear anxiety fear fear fear fear fear fear sadness depressive sadness alone guilt happy distress distress irritability panic stress relieve overwhelming loved*
101,019	0.06	0.03	*happy alone afraid grateful sorrow triumphs proudest desperately content peacetime peace peacefulness hope happy entertainment hope satisfying surprise joy interesting yearns peace afraid regrets proudest love appreciation appreciation hope proud peace secure happier bad bad bad awful pleasure hopefuls hopeful hope triumph proud passion surprise hope enthusiasm bad shocked outrageous surprise dreading passions pleasure joy happiness happy happiness happiness joy interests passions happiness joy happiness love loved hated hated satisfaction enjoy enjoy joy joy hate joy happiness enjoy enjoy admiration affection loved bad bad embarrassing regret worrisome happy content interest interest interest satisfying satisfying merry-go-round frustrating dismayed interest worry worried hope bad discourage enjoy passion afraid afraid proud proud entertaining pleasure love discourages afraid afraid exciting fear interested love interest enjoy enjoy alone enjoy thankful happy happy triumph sadly cherished frustrated alone desperate happy loves love hurt hurt love hurt alone surprise hurt love loves love love love enjoy admiration affection loved bad bad embarrassing enjoy love pleasure joy love miss joy alone love excited bad s worry cheerful enjoy grieve love cherish guilt guilt love love hope unhappy hurt loved love humiliation love unhappy happiness love love secure irritations offense bad sad glad loving love peace love hate fear content yearn love loved joy entertainment jealous alone joy love afraid afraid afraid afraid fear love peace worry depression stress panic thankful happy contented love affection scared scared delighted upset worry startled glad alarm frustrations irritations aggravations terror crazy alarm offence frustrations irritations aggravations peacefully amused crap afraid upset alone bad love love pity love love restless excitement relief s afraid secure exciting surprise envy joy loving loving emotional depression frustration emotional depression emotional interested embarrassment depressed emotional depressed emotional emotional love resolved guilt-free passionate interest stress stress overwhelming distress loss emotional depression emotional nervous stress loss panic panic emotional doom alarm panic emotional panic panic loneliness depression happy resolve crap embarrassing interesting delighted passionately frantic horribly happily happy hatred worries appreciate crap agony shock resolve resolve anger frustrations stressful fear stress enjoyable*

			interested enjoys love pleasure happy enjoy pleasure passionate love love thrill love pleased embarrassed furious satisfying passion fondness pleasure surprising delightful bad admire love enjoying love enjoy enjoy admired satisfy interested love enjoys enjoy love worry hurt panic worry joy loss loss satisfying fear peace love hope enthusiasm worry joy loss loss satisfying fear peace love hope enthusiasm shock love loves love miss loved crazy love love anxious love depression loss nervous happiness surprise interesting hope fearing alone love interested sadly depressed peace loving dislike love loved adorable passion love love peace loved love love cheerful cheer dislike love love love love depressed peace loving dislike love loved adorable passion love love peace loved love love cheerful cheer dislike love love love love love enjoyable depression crazy tranquilizer relieves dreaded passionately stress love joys love content love love alone love alone horribly happily happy hatred worries appreciate crap agony shock interested satisfied enjoy happy affection affection appreciate affection passionate jealous jealous interested hate enjoy passionate love affectionate satisfy passion loved appreciated enjoy pleasures interested satisfied thrill passionate love love loving appreciated emotional passionate enjoy love interested passionate passions enjoy enthusiastic pleasure jealous love love love enjoy enthusiastic happiest love adore excitement enjoy excitement fear proud love love love love love pleasures love interesting amuses appreciative irritated pleasant admire exciting enjoys enjoy excitement amusing interesting cheer surprise nervous worrier s secured hurt terrible interesting love entertaining love afraid passion interested love disappointed afraid intimidating content love fears worry scared desperate passionate afraid lone interest lone depressed peace loving dislike love loved adorable passion love love peace loved love love cheerful cheer dislike love love love love afraid surprised excited stressed interested hope admired surprised entertained pleasant pleased admired yearned exciting hope admired surprised entertained pleasant pleased admired yearned exciting depressed peace loving dislike love loved adorable passion love love peace loved love love cheerful cheer dislike love love love love scared enjoyed admiring horrendous scared worried relieved joy surprised glad interesting enjoyed fear interesting enjoyed bad regrets enjoying regrets worries afraid exciting passionate amusement appreciated thrilled adorable hope admired surprised entertained pleasant pleased admired yearned exciting interesting interesting interesting glad grateful enjoyed terribly hope glad securing security scary love love loving yearn yearning disheartened love shocking disappointment hope love love worries annoying enjoying loves satisfied anxious love alone love love excitement love peace love hope enjoy love surprises loneliness agony fear thankful loss happiness irritated frustrated thankful happy contented shocked crazy worrying overwhelming shocked unhappily unhappy madder madder angrily surprised unpleasant irritate angry hurt bitter jealous hurt angry bitter agony unpleasant hurt unpleasant bad miss love alarm desperately surprise depressing enjoy thankful affection appreciation appreciate

			interesting disappointed satisfaction resolve resolve anger frustrations stressful fear stress enjoyable entertainment jealous terrible bad bad bad bad happier desperately surprise passions-things happiness glad surprise love love sad love love love love love admired love adored love angry nervous relieved surprised terribly nervous hopeful fear disturbance fear fear panic devastating devastating passions insecurity anger apathy fear worry bad regret love enthusiasm passionate proud appreciate hate hating happy love hurt horrible love bad loves love love love appreciate love love love love love love love love joys love love love love love love love love love love love love love love love love love glad loved love love happy love loves bad bad bad bad bad bad bad alone worry joy loss loss satisfying fear peace love hope enthusiasm interesting stress stress frustration hurt hurt appreciate love depressing enjoy jealous jealous happiness love loving joyous happy happy glad regretfully love love love hurt relief emotional love love joy happy happy happy shock bad bad terrible hope bad resolve resolve anger frustrations stressful fear stress enjoyable loves admirer terribly terrible bad bitter crushed emotional regrets love bad sad love love love love love love love love worried enjoying jealous hate jealous jealous proud hopeless love love jealous love love love restless enthusiasm regrets love interested love love love love love love love jealous love love love love jealous love love anger jealous peace love love love bad love love hope hope love love love discouraged hurt upsetting hurt hurt hurt hurt content yearn love loved joy bad interesting depressed admirer enjoy entertaining entertainment entertaining interesting hate alone miss miss miss hate love passionate emotional emotional jealousy emotional enjoy worry enjoy hope secure worried secure interesting interesting terribly fear scared scared excited afraid security lonely reluctantly love content love love alone insecure scared insecure secure alone scared loved love love bad worry afraid afraid agitatedly irritation angrily cheerful disgust solemnly love happiness happiness adorable happiness happiness happy anger worry worry frustration happiness lonely happier happier happy happier triumphantly happier happiness happy love bad depression numb emotional hurt numb happiness love lonely lonely jealous

Supplementary Table 2. Pearson Correlations Between Text-Derived and Self-Reported Individual Difference Variables (Study 1)

	Individual Differences—Text-derived					
	Illness words	I-words	We-words	Affiliation words	Achievement words	Leisure words
Personality variables[a]						
Openness	.027	-.116***	.041	-.004	-.003	.024
Conscientiousness	.015	-.071**	.112***	.090**	.093**	.021
Extraversion	.067*	.008	.032	.084**	.028	.012
Agreeableness	-.017	.012	.004	.066*	.111***	.049
Neuroticism	.069*	.189***	-.129***	-.072**	-.022	-.116***
Depression symptoms[b]	.065*	.121***	-.120***	-.077**	.000	-.074**
Overall health[c]	-.010	-.084**	.070**	.068**	.050*	.087**

*** $p < .001$. ** $p < .01$. * $p < .05$. All correlations were two-tailed.

[a] N= 1,341 based on available data.

[b] N= 1,256 based on available data.

[c] N= 1,545 based on available data.

Supplementary Note 1. Exploration of Interactive Effects of Positive and Negative EV

Although not a primary aim of the present report, it is interesting to consider to potential effects of co-occurring broad vocabularies in predicting well-being. Using a multiple regression technique, we examined the effect of the interaction of Positive and Negative EV predicting self-reported depression scores, controlling for both EV main effects and the three covariates used in the manuscript's partial correlations (negative and positive emotional tone and general vocabulary). Results revealed a significant moderation suggestive of a buffering effect of positive emotion vocabularies, b=-.84, SE=.40, p=.035, with this interaction accounting for 3.4% of depression variance. As shown in the figure below, the pattern of effects was such that for individuals with very narrow negative emotion word repertoires, depression symptoms were lower and did not depend on Positive EVs. By contrast, for individuals with broad Negative EVs, there was a buffering effect of Positive EV, such that individuals with restricted Positive EV were more depressed. Interestingly, however, this buffering effect reached significance only at exceedingly high levels of Negative EV (i.e., above values of 1.27, or above the 97.3rd percentile).

This moderation finding appears to suggest that possessing and/or using a varied positive emotion vocabulary mitigates the relationship between varied negative vocabularies and depression, but given the correlational design, we cannot comment on the causality in this effect. A more descriptive interpretation, consistent with literature on depressive cognition (e.g., Nolen-Hoeksema, 1991), is that depression symptoms are associated with repertoires for naming emotion that are *simultaneously* relatively well developed for negative emotions *and* impoverished for naming positive emotions.

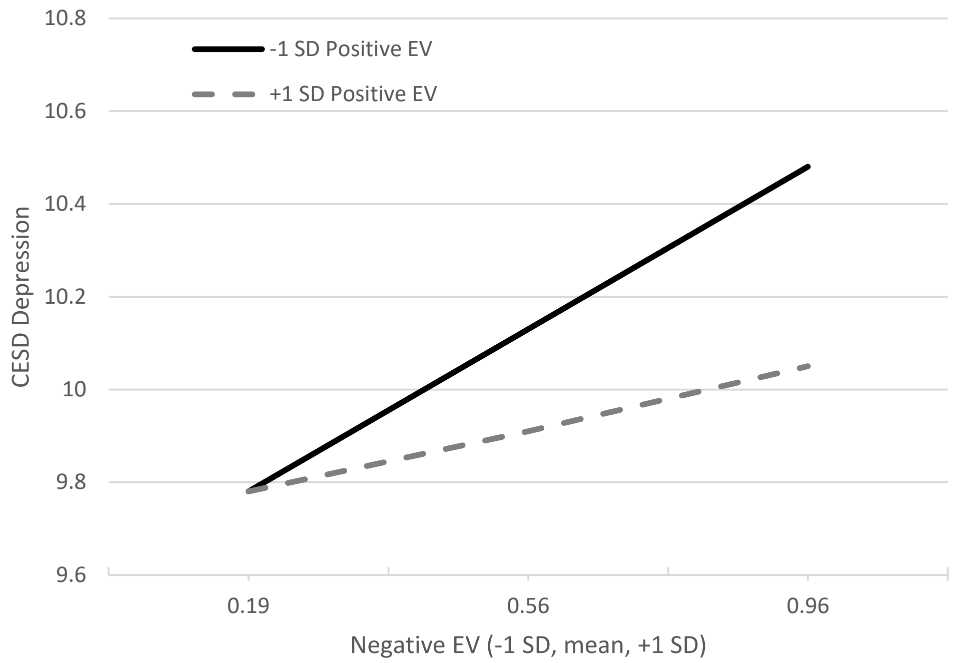

Supplementary Figure 1. Interactive effects of Positive EV as a moderator of the relationship between Negative EV and depression symptoms. Consistent with the approach in the main manuscript, covariates included in the model are negative and positive emotional tone and general (i.e., non-emotion) vocabulary. Values for +/-1 SD for Negative EV appear on x-axis; values of +/-1 SD for Positive EV are .86 and .18, respectively. Source data are provided as a Source Data file.

Supplementary Table 3. **Age of Acquisition (AoA) norms for emotion words, drawn from Kuperman and colleagues**[1]

Vocabulate Dictionary Item	Neg	Pos	Closest word form in AoA corpus	Mean AoA	SD of AoA	-1 SD age	+1 SD age
afraid	X		afraid	4.42	1.71	2.71	6.13
aggravat*	X		aggravated	8.61	2.18	6.43	10.79
agitat*	X		agitated	10.12	4.44	5.68	14.56
agony	X			9.22	2.65	6.57	11.87
alarm*	X			6.39	2.68	3.71	9.07
alone	X		alone	4.94	1.73	3.21	6.67
anger|angr*	X		anger	6	2.58	3.42	8.58
anguish*	X		anguish	11.54	3.18	8.36	14.72
annoy*	X		annoyed	7.72	2.97	4.75	10.69
anxi*	X		anxious	10.21	2.53	7.68	12.74
apath*	X		apathy	14.21	3.22	10.99	17.43
appall*	X		appalled	11.53	3.87	7.66	15.4
apprehens*	X		apprehensive	11.45	3.05	8.4	14.5
asham*	X		ashamed	7.11	3.7	3.41	10.81
aversi*	X		aversion	11.94	3.4	8.54	15.34
bitter	X		bitter	8.81	3.7	5.11	12.51
contempt	X		contempt	12.44	2.73	9.71	15.17
crushed	X						
depress*	X		depressed	9.47	2.44	7.03	11.91
despair	X		despair	9.56	2.94	6.62	12.5
desperate*	X		desprate	9.22	2.67	6.55	11.89
despis*	X		despise	12.38	2.64	9.74	15.02
devastat*	X		devastation	10.83	2.96	7.87	13.79
disappoint*	X		disappointed	6.95	2.68	4.27	9.63
discourage*	X		discouragement	10.22	3.05	7.17	13.27
disgust	X		disgust	7.68	3.37	4.31	11.05
dishearten*	X		dishearten	11.22	2.18	9.04	13.4
disillusion*	X		disillusionment	13.95	3.05	10.9	17
dislike […]	X		dislike	5.42	2.36	3.06	7.78
dismay*	X		dismay	10.9	3.02	7.88	13.92
dissatisf*	X		dissatisfied	9.95	3.05	6.9	13
distraught	X		distraught	11	2.45	8.55	13.45
disturb*	X						
doom	X		doom	9.42	3.22	6.2	12.64
dread*	X		dread	9.44	2.73	6.71	12.17
embarrass*	X		embarrassed	7.83	2.64	5.19	10.47
envy […]	X		envy	8.37	2.95	5.42	11.32
fear […]	X		fear	4.79	1.93	2.86	6.72
frantic	X		frantic	10.67	2.17	8.5	12.84
fright	X		frightened	5.65	3.13	2.52	8.78
frustrat*	X		frustrated	7.97	2.63	5.34	10.6
fury|furious	X		furious	8.78	2.94	5.84	11.72
gloom	X		gloom	9	1.69	7.31	10.69
grief|griev*	X		grief	8.39	2.62	5.77	11.01

guilt*	X		guilt	7.05	2.57	4.48	9.62
hate […]	X		hate	5.53	2.06	3.47	7.59
heartbreak* […]	X		heartbreak	10.32	2.89	7.43	13.21
helpless	X		helpless	8.32	3	5.32	11.32
homesick	X		homesick	8.83	3.43	5.4	12.26
hopeless	X		hopeless	8.2	1.82	6.38	10.02
hostil*	X		hostile	9.9	2.66	7.24	12.56
humiliat*	X		humiliating	9.89	2.31	7.58	12.2
hurt	X		hurt	4	1.45	2.55	5.45
insecur*	X		insecure	12.11	3.65	8.46	15.76
intimidat*	X		intimidating	10.47	3.24	7.23	13.71
irrita*	X		irritable	8.67	3.09	5.58	11.76
jealous*	X		jealous	7.42	2.58	4.84	10
lone*	X		lonely	5.8	2.35	3.45	8.15
longing	X						
loss	X		loss	5	2.38	2.62	7.38
mad […]	X		mad	3.55	1.5	2.05	5.05
melanchol*	X		meloncholy	12.53	4.33	8.2	16.86
miss	X		miss	6.33	3.41	2.92	9.74
mourn	X		mourn	9.21	2.97	6.24	12.18
nervous	X		nervous	6.79	2.35	4.44	9.14
offend […]	X		offend	9.67	3.48	6.19	13.15
outrag*	X		outrage	9.9	2.13	7.77	12.03
panic	X		panic	8.35	2.98	5.37	11.33
paranoi*	X		paranoid	12.3	3.16	9.14	15.46
petrif*	X		petrified	9.05	2.37	6.68	11.42
phobi*	X		phobia	9.83	3.24	6.59	13.07
pity	X		pity	8.72	3.44	5.28	12.16
rage	X		rage	8.79	3.21	5.58	12
regret*	X		regret	9.61	2.38	7.23	11.99
reluctan*	X		relunctant	11.11	3.05	8.06	14.16
remorse*	X		remorse	11.11	2.45	8.66	13.56
resent*	X		resent	11.22	3.08	8.14	14.3
restless	X		resless	9.74	2.92	6.82	12.66
sad […]	X		sad	3.24	2.21	1.03	5.45
solemn*	X		solemn	12.5	3.2	9.3	15.7
scared […]	X		scared	3.79	1.47	2.32	5.26
ashame*	X		ashamed	7.11	3.7	3.41	10.81
shock*	X		shock	7.53	2.32	5.21	9.85
sorrow	X		sorrow	8.42	2.61	5.81	11.03
startl*	X		startle	9.17	2.5	6.67	11.67
stunned	X		stun	9.56	2.85	6.71	12.41
terror […]	X		terror	8.79	3.77	5.02	12.56
turmoil	X		turmoil	12.84	3.4	9.44	16.24
unhapp*	X		unhappiness	6.84	2.46	4.38	9.3
unlov*	X		unloved	7.28	1.93	5.35	9.21
woe*	X		woe	10.75	3.27	7.48	14.02
worr*	X		worried	6.65	2.39	4.26	9.04
yearn*	X		yearn	11.65	3.34	8.31	14.99
awful			awful	6.26	2.51	3.75	8.77
bad			bad	2.79	1.27	1.52	4.06

crap|crappy*			crap	6.5	2.57	3.93	9.07
craz*			crazy	5.22	1.66	3.56	6.88
distress*			distress	11.16	3.93	7.23	15.09
emotional			emotional	9.45	4.57	4.88	14.02
horr*			horrible	5.81	2.66	3.15	8.47
rotten			rotten	6.95	2.33	4.62	9.28
numb […]			numb	9.26	3.93	5.33	13.19
overwhelm*			overwhelming	9.94	2.71	7.23	12.65
stress*			stress	10	3.25	6.75	13.25
sucky			suck	5.58	2.39	3.19	7.97
terribl*			terrible	5.56	1.92	3.64	7.48
unpleasant			unpleasant	9.32	3.43	5.89	12.75
upset*			upset	5.26	2	3.26	7.26
admir*		X	admire	7.42	2.14	5.28	9.56
ador*		X	adore	7.95	3.31	4.64	11.26
affection*		X	affection	8.53	2.97	5.56	11.5
amor*		X	amorous	13.71	2.71	11	16.42
amus*		X	amused	7.83	3.17	4.66	11
appreciat*		X	appreciate	8.8	3.19	5.61	11.99
cheer […]		X	cheerful	7.68	2.87	4.81	10.55
cherish*		X	cherish	8.9	2.61	6.29	11.51
content […]		X	contentment	10.72	3.12	7.6	13.84
deligh*		X	delight	8.68	2.65	6.03	11.33
ecsta*		X	ecstatic	13.26	3.6	9.66	16.86
enjoy*		X	enjoy	5.75	2.17	3.58	7.92
entertain*		X	entertaining	8.84	2.61	6.23	11.45
enthus*		X	enthusiastic	9.17	2.12	7.05	11.29
excit*		X	excited	6.21	1.69	4.52	7.9
fondness		X	fond	9.21	2.42	6.79	11.63
glad|gladly		X	glad	5.79	2.18	3.61	7.97
gratef*		X	grateful	7.42	2.48	4.94	9.9
happy|happi*		X	happy	2.72	1.6	1.12	4.32
hope […]		X	hope	4.89	2.11	2.78	7
interest*		X	interested	8.83	2.62	6.21	11.45
joll*		X	jolly	6.59	2.03	4.56	8.62
joy*		X	joy	6.74	2.92	3.82	9.66
keen*		X	keen	10.7	1.56	9.14	12.26
love […]		X	love	5.17	3.54	1.63	8.71
merr*		X	merry	5.89	3.1	2.79	8.99
passion*		X	passion	10.79	3.9	6.89	14.69
peace*		X	peace	6.32	2.52	3.8	8.84
pleased […]		X	pleasure	8.21	2.37	5.84	10.58
proud*		X	proud	5.44	1.58	3.86	7.02
relief|reliev*		X	relief	7.79	1.44	6.35	9.23
resolv*		X	resolve	11	3.69	7.31	14.69
satisf*		X	satisfied	9.68	3.2	6.48	12.88
secur*		X	secure	8.42	2.41	6.01	10.83
surpris*		X	surprised	6.63	2.87	3.76	9.5
thankf*		X	thankful	5.5	1.64	3.86	7.14
thrill*		X	thrill	8.37	2.17	6.2	10.54
tranquil*		X	tranquil	13.42	3.53	9.89	16.95

triumph*		X	triumphant	8.89	3.3	5.59	12.19
vigor […]		X	vigor	10.63	2.99	7.64	13.62

Note. For more complete information on the Vocabulate dictionary items (left column) see also Supplementary Table 1. Long items are abbreviated in this table with "[…]" for spacing reasons.

Supplementary Note 2. Variations in Emotion Vocabulary as a Function of Writing Topic

It is reasonable to assume that the use of emotion-related language will vary from topic to topic – for example, a person may use more emotional language when talking about a recent romantic breakup or illness than when discussing the usefulness of various building materials. In order to preliminarily explore the degree to which Emotion Vocabularies may vary across content and contexts, we used the meaning extraction method (MEM) topic modeling approach[2-4] to extract and quantify topics from each study in a data-driven manner. Unlike the LIWC categories used in the present study, which are determined *a priori*, MEM themes are derived in a bottom-up fashion from the emergent language patterns of the texts. By reducing the semantic dimensionality of words used in each corpus, we established and subsequently quantified the overarching "themes" or "topics" present in the writing samples from each study, respectively, which could then be statistically examined for their relationships to participant EVs.

We evaluated the correlations between MEM topic scores and EV scores. As shown in Supplementary Table 4, below, there were clear patterns of correlation, such that broader EVs were generally correlated in interesting and often intuitive ways with various topics. For example, in Study 1, students who more prominently invoked the topic of college (characterized by high loadings of the words "year," "campus," "college," "degree," "student," "major") used less diverse negative EVs (**r** = -.08, p=.001) and more diverse positive EVs (**r** =.15, p<.001). Students invoking the topic of sleep ("day," "early," "exhaust," "late," "hour," "nap," "bed") used less diverse positive EVs (**r** = -.13, p<.001). In Study 2, bloggers who more frequently showed a poetic theme ("heart," "soul," "eye," "tear," "darkness," "deep," "light," "dream," "sky") used more diverse emotion vocabularies of both valences, while bloggers who wrote more on the theme of recipes ("pepper," "recipe," "butter," "salad," "tomato," "cut," "stir") used less

diverse EVs of both valences. These results echo the primary findings in the manuscript, in that they give an impression of diversity in emotion language mirroring concerns with themes germane to distress and wellbeing.

However, it is important to note that the MEM themes are corpus-specific and, therefore, we cannot definitively state which themes may be diagnostic of other objective measures of wellbeing. We therefore must necessarily consider them to have only tentative explanatory value in this context with respect to our primary research questions, unlike the LIWC categories which have been demonstrated to be valid, highly reliable indicators of wellbeing and physical health across hundreds (if not thousands) of previous empirical studies. More extensive analysis of the topical data is thus beyond the scope of the present manuscript, which strives to examine the relationship of emotion vocabulary breadth to distress/wellbeing. In the future, more computationally sophisticated approaches such as the MEM and semantic vector models will be useful in future work for unpacking and establishing how different emotion words may show concept-specific associations with various topical domains.

Supplementary Table 4. Variations in Emotion Vocabulary as a Function of Writing Topic Expressed as Pearson Correlation Coefficients

MEM Theme	Characteristic Words	Negative EV	Positive EV
	**Study 1: Stream of Consciousness Essays (Time 1 only)**		
Writing prompt	*type, minute, write, assignment, spell, thought, random, stream, hmm, consciousness, text*	-.10***	-.11***
Classes/exams	*chemistry, test, exam, study, class, benchmark, biology, quiz, note, tomorrow, material, week, homework*	-.30***	-.41***
Academic success	*major, business, degree, job, graduate, student, future, apply, program, important, gpa, life, career*	-.09***	.06*
Dating/friends	*relationship, friend, emotion, talk, best, happy, boyfriend, met, person, together, guy, care*	.12***	.05*
Family	*family, parent, sister, austin, house, mom, year, brother, dad, visit, car, school, town,*	.14***	.11***
Social clubs	*group, join, club, meet, meeting, organization, sorority, involve, friend, event*	-.02	.00
Weekend/plans	*saturday, birthday, dinner, today, weekend, sunday, lunch, tomorrow, friday, tonight*	-.09***	.01
Sleep	*wake, sleep, night, morning, asleep, tire, nap, bed, exhaust, fall*	-.06*	-.15***
Poetic	*story, grow, beautiful, face, heart, remember*	-.20***	-.02
Balance/stress	*balance, figure, focus, stress, social, constantly, overwhelm, huge*	-.07**	.05*
Entertainment	*game, football, show, play, team, watch, tv, practice, video, band*	-.20***	.01
Diet/body	*weight, eat, gain, gym, food, healthy, body, habit, hungry, lose*	.05	.01
Listening to music	*music, song, listen, play, band, mood, favorite, hear, remind*	-.01	.12***
Roommates	*room, outside, roommate, quiet, apartment, awkward, comfortable, loud, alone, move, clean*	.06*	-.12***
	**Study 2: Blogs**		
Existential	*individual, human, experience, society, existence, provide, knowledge, understanding, sense, belief*	-.04***	.05***
Poetic	*heart, soul, eye, tear, pain, darkness, deep, light, inside, dream, sky, hold, fade*	.30***	.09***
Computers	*user, microsoft, window, file, install, server, software, system, version, data, download, application*	-.10***	-.05***
Presidential election	*republican, bush, kerry, democratic, democrat, campaign, president, election, vote*	-.05***	-.05***
Iraq War	*Iraq, Iraqi, war, military, soldier, saddam, weapon, attack, terrorist, troop*	-.00	-.07***
Cooking/food	*pepper, recipe, butter, salad, tomato, onion, sugar, cut, stir, slice*	-.06***	-.02*
Religion	*god, jesus, christ, mercy, lord, pray, church, prayer, christian, bible, worship, spirit*	-.03**	.01
Business	*develop, business, management, company, technical, design, manager, customer*	-.06***	-.05***
Sports	*team, win, league, player, game, season, baseball, play, score, pitch*	-.06***	-.03*
Sensual	*mouth, tongue, hand, gently, lip, body, eye, moan, slowly, gasp*	-.04***	-.05***
Nature	*mountain, river, island, beach, north, lake, trip, hike, ride, sea, trail*	-.13***	.00
Music	*music, band, song, album, guitar, drum, bass, vocal, rock, listen, record*	-.08***	.01
Finances	*pay, tax, loan, money, income, expense, payment, saving, cost, fee, rate, insurance*	-.06***	-.04***
Work/life	*job, work, life, find, time, life, place, change, money, year, child, career, small, marry*	.03**	.10***
Film	*movie, film, actor, scene, character, comedy, watch, plot*	-.07***	.03**
Weekends	*weekend, week, friday, work, saturday, sunday, monday*	.02*	.24***
Education	*school, student, teacher, class, classroom, teach, grade, college, study, education*	-.04***	.01
Home Renovation	*room, kitchen, floor, bedroom, bathroom, carpet, wall, paint*	-.04***	-.07***
Evolution	*species, universe, human, earth, planet, scientist, evolve, natural, existence, theory*	-.11***	-.10***

Note. Table shows strongest MEM themes for each sample (i.e., presence of loadings > .3 and interpretable theme). Correlations are two-tailed. *p<.05. **p<.01. ***p<.001.

Supplementary Note 3. Negligible Impact of Word List Length, Balance, and Composition

Readers may wonder about the implications of the particular composition of the word lists in the EV, including the effect of the imbalance between the numbers of negative and positive words (92 vs. 53). In the abstract, it would seem that a measurement tool should have a ceiling high enough to detect vocabulary variability in higher ranges, and this ceiling should be comparable between positive and negative valences. Such a criticism regarding ceilings imposed by instruments has been leveled convincingly about a non-linguistic index of diversity in self-reported emotion experience[5]. Practically, however, in the context of a word-counting approach, the effect of the word list length becomes inconsequential. English, like other languages, contains more negative than positive words in their lexica[6,7]. Highly nuanced words for positive emotional states are used so rarely in natural speech, that the upper ranges of vocabulary counts will likely not be reached in a natural language sample. For example, in the student sample (Study 1) the majority of words counted (74%) were used by fewer than 1% of students, and 25% were only used once in the entire corpus. Put another way, the more sophisticated word "enthralled" occurred in < 0.5% of the blogs (Study 2) and not at all in the student writing (Study 1). By comparison "love," "happy," and "excited" occurred, respectively, in 65%, 47%, and 22% of blogs, and 46%, 35%, and 24% of student texts. Although raising the positive word list to 92 items might equalize the ceiling for possible positive and negative EV scores, in practice this would not affect sample-wide relationships. In this way, word-counting-based approaches, while imprecise for individual-level diagnostic assessment, are robust against measurement noise at the group level.

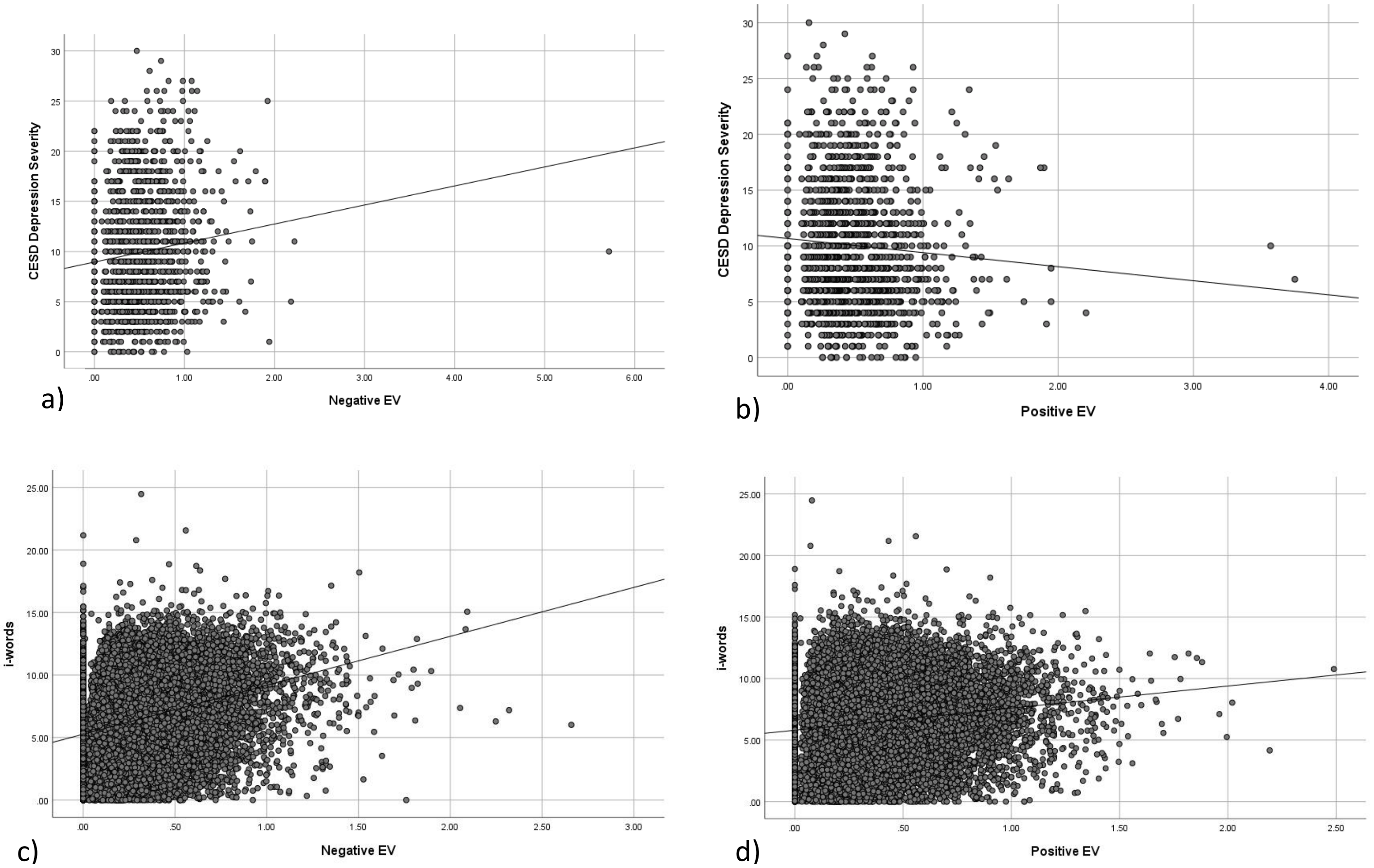

Supplementary Figure 2. Scatterplots for Visualizing Study Data. (a) Negative EV scores and self-reported depression symptoms (CESD) from Study 1; (b) Positive EV scores and self-reported depression symptoms (CESD) from Study 1; (c) Negative EV scores and text-derived index (*i-words*) from Study 2; (d) Positive EV scores and text-derived index (*i-words*) from Study 2. Source data are provided as a Source Data file.

Supplementary Table 5

95% Confidence Intervals and Exact Significance Levels for Pearson and Partial Correlations of Emotion Vocabulary (EV) with Other Study Variables for Study 1 (N = 1,567 unless marked otherwise)

	Pearson Correlations with Negative EV	Partial Correlations with Negative EV	Pearson Correlations with Positive EV	Partial Correlations with Positive EV
Negative EV	--	--	--	--
Positive EV	.16 [.05, .27], *p* < .001	.18 [.10, .24], *p* < .001	--	--
**Demographic variables**				
Age	-.02 [-.07, .02], *p* = .388	-.05 [-.10, -.01], *p* = .050	-.06 [-.09, -.02], *p* = .022	-.04 [-.08, .00], *p* = .118
Gender[a]	.20 [.02, .24], *p* < .001	.15 [.10, .20], *p* < .001	-.00 [-.05, .06], *p* = .967	.06 [.01, .11], *p* = .024
**Individual differences—text-derived**				
Cognitive processing	.08 [.03, .13], *p* = .002	.06 [.01, .12], *p* = .014	-.07 [-.13, -.01], *p* = .007	-.02 [-.08, .04], *p* = .365
Negative emotional tone	.61 [.54, .69], *p* < .001	--	.01 [-.09, .13], *p* =.731	--
Positive emotional tone	-.03 [-.10, .05], *p* = .297	--	.50 [.45, .55], *p* < .001	--
General vocabulary size	.11 [.04, .17], *p* < .001	--	.21 [.15, .26], *p* < .001	--
Illness words	.11 [.03, .19], *p* < .001	.01 [-.04, .07], *p* = .603	.06 [-.01, .13], *p* = .027	.06 [.00, .11], *p* = .031
I-words	.25 [.10, .36], *p* < .001	.23 [.14, .32], *p* < .001	.05 [-.05, .13], *p* = .062	.10 [.03, .18], *p* < .001
We-words	-.11 [-.15, -.08], *p* < .001	-.06 [-.10, -.01], *p* = .026	-.05 [-.10, .01], *p* = .048	-.06 [-.12, -.01], *p* = .012
Affiliation words	.05 [-.01, .10], *p* = .058	.09 [.04, .14], *p* < .001	.14 [.07, .23], *p* < .001	.06 [.00, .11], *p* = .027
Achievement words	.06 [-.04, .15], *p* = .031	.04 [-.03, .11], *p* = .094	.11 [.04, .18], *p* < .001	.00 [-.06, .06], *p* = .948
Leisure words	-.07 [-.17, .03], *p* = .004	-.02 [-.10, .05], *p* = .342	.20 [.10, .30], *p* < .001	.08 [-.01, .15], *p* = .002
**Individual differences—self-reported**				
Openness[b]	-.03 [-.09, .03], *p* = .323	-.02 [-.08, .03], *p* = .494	.04 [-.01, .10], *p* = .162	.01 [-.05, .06], *p* = .787
Conscientiousness[b]	-.01 [-.07, .06], *p* = .982	.06 [.01, .12], *p* = .032	.06 [.01, .12], *p* = .035	.07 [.01, .12], *p* = .026
Extraversion[b]	-.04 [-.09, .01], *p* = .186	-.03 [-.09, .03], *p* = .298	.06 [.01, .12], *p* = .035	.03 [-.02, .09], *p* = .268
Agreeableness[b]	.01 [-.05, .06], *p* = .835	.05 [-.01, .10], *p* = .075	.09 [.03, .15], *p*= .002	.06 [-.01, .11], *p* = .050
Neuroticism[b]	.17 [.12, .23], *p* < .001	.08 [.02, .15], *p* = .009	-.09 [-.14, -.03], *p* = .003	-.02 [-.08, .04], *p* = .545
Depression symptoms[c]	.11 [.06., .17], *p* < .001	-.01 [-.07, .06], *p* = .802	-.07 [-.12, -.01], *p* = .024	-.01 [-.07, .05], *p* = .716
Overall health[d]	-.13 [-.18, -.08], *p* < .001	-.05 [-.11, .00], *p* = .068	.06 [.01, .11], *p* = .041	.05 [.04, .15], *p* = .109

Note. Partial correlations control for general vocabulary, and negative and positive emotional tone. All tests are two-tailed.

Coefficients are expressed as **r** [95% CI], based on 2,000 bias corrected and accelerated bootstrapped replicates with replacement.

[a] Coded 0 = male, 1 = female.

[b] n = 1,341 student essays based on available data.

[c] n = 1,256 student essays based on available data.

[d] n = 1,545 student essays based on available data.

Supplementary Table 6

95% Confidence Intervals and Exact Significance Levels for Partial Correlations between Emotion Vocabulary (EV) for Distinct Emotion Types and Changes in Self-Rated Moods in Study 1 (N = 1,546)

Emotion Vocabulary	Sadness mood change	Worry mood change	Anger mood change	Stressed mood change	Positive mood change
Sadness	.09 [.04, .15], $p < .001$	.02 [-.03, .07], $p = .487$	-.08 [-.12, -.03], $p = .003$	.01 [-.05, .06], $p = .793$	-.01 [-.06, .03], $p = .581$
Fear	-.03 [-.08, .02], $p = .184$	.09 [.03, .14], $p < .001$	-.12 [-.16, -.08], $p < .001$	.02 [-.04, .08], $p = .550$	.06 [.01, .11]], $p = .013$
Anger	.01 [-.03, .06], $p = .577$	.05 [-.01, .10], $p = .080$	.10 [.05, .15], $p < .001$	.05 [.00, .10], $p = .064$	-.10 [-.15, -.04], $p < .001$
Neg Undiff	.00 [-.05, .04], $p = .941$	.06 [.01, .11], $p = .019$	-.01 [-.06, .04], $p = .626$	.09 [.04, .13], $p = .001$	-.02 [-.06, .03], $p = .485$
Positive	-.04 [-.09, .01], $p = .104$	.02 [-.03, .08], $p = .355$	-.07 [-.12, -.03], $p = .005$	-.02 [-.08, .03], $p = .391$	.04 [.00, .09], $p = .099$

Note. Values are partial correlation coefficients between EV indices and post-writing ratings of subjective mood. Each correlation controls for pre-writing levels of the target mood, as well as general vocabulary, and negative and positive emotional tone. Sample size is based on availability of state mood ratings. Neg Undiff = negative undifferentiated words. All tests were two-tailed. Coefficients are expressed as **r** [95% CI] based on 2,000 bias corrected and accelerated bootstrapped replicates with replacement.

Supplementary Table 7

95% Confidence Intervals and Exact Significance Levels for Pearson Correlations of Emotion Vocabulary (EV) with Other Study Variables for Study 2 (N = 35,385)

	Pearson Correlations with Negative EV	Partial Correlations with Negative EV	Pearson Correlations with Positive EV	Partial Correlations with Positive EV
Negative EV	--	--	--	--
Positive EV	.22 [.20, .23], $p < .001$	.12 [.09, .15], $p < .001$	--	--
**Demographic variables**				
Age[a]	-.09 [-.10, -.01], $p < .001$	.01 [-.02, .03], $p = .647$	.05, [.02, .07], $p < .001$	-.07 [.04, .09], $p < .001$
Gender[b]	.15 [.13, .17], $p < .001$	.15 [.13, .17], $p < .001$	.07 [.05, .09], $p < .001$	.07 [.05, .09], $p < .001$
**Individual differences—text-derived**				
Cognitive processing	.21 [.20, .22], $p < .001$	--	.08 [.07, .09], $p < .001$	--
Negative emotional tone	.51 [.49, .52], $p < .001$	--	-.03 [-.04, 0.02], $p < .001$	--
Positive emotional tone	.09 [.07, .10], $p < .001$	--	.35 [.34, .36], $p < .001$	--
General vocabulary size	.24 [.23, .25], $p < .001$	--	.46 [.46, .47], $p < .001$	--
Illness words	.16 [.15, .18], $p < .001$	.07 [.05, .08], $p < .001$	.07 [.06, .08], $p < .001$	.06 [.04, .07], $p < .001$
I-words	.28 [.27, .29], $p < .001$	.20 [.18, .21], $p < .001$	.13 [.12, .14], $p < .001$	.10 [.08, .11], $p < .001$
We-words	-.08 [-.09, -.07], $p < .001$	.00 [-.01, .01], $p = .502$	-.02 [-.03, -.01], $p = .005$	.00 [-.02, .01], $p = .421$
Affiliation words	-.01 [-.02, .01], $p = .327$	.06 [.05, .07], $p < .001$	.08 [.07, .10], $p < .001$	.03 [.02, .04], $p < .001$
Achievement words	-.10 [-.11, -.09], $p < .001$	-.07 [-.09, -.06], $p < .001$	.06 [.05, .07], $p < .001$	-.01 [-.02, .01], $p = .301$
Leisure words	-.14 [-.15, -.13], $p < .001$	-.09 [-.10, -.08], $p < .001$	.06 [.04, .07], $p < .001$	-.05 [-.06, -.04], $p < .001$

Note. Partial correlations control for general vocabulary, and negative and positive emotional tone. All tests are two-tailed. Coefficients are expressed as **r** (SE), based on 2,000 bias corrected and accelerated bootstrapped replicates with replacement.

[a] For analyses involving age, n = 9,805 blogs.

[b] Coded 0=male, 1=female.

Supplementary References

1. Kuperman, V., Stadthagen-Gonzalez, H. & Brysbaert, M. Age-of-acquisition ratings for 30,000 English words. *Behav. Res. Methods* **44**, 978-990 (2012).
2. Argamon, S., Koppel, M., Pennebaker, J. W. & Schler, J. Mining the blogosphere: Age, gender, and the varieties of self-expression. *First Monday* **12** (2007).
3. Boyd, R. L. & Pennebaker, J. W. Language-based personality: A new approach to personality in a digital world. *Curr. Opin. Behav. Sci.* **18**, 63–68 (2017).
4. Chung, C. K. & Pennebaker, J. W. Revealing dimensions of thinking in open-ended self-descriptions: An automated meaning extraction method for natural language. *J. Res. Pers.* **42**, 96-132 (2008).
5. Brown, N. J. & Coyne, J. C. Emodiversity: Robust predictor of outcomes or statistical artifact? *J. Exp. Psych. Gen*. **146**, 1372-1377 (2017).
6. Bednarek, M. *Emotion Talk Across Corpora*. (Palgrave, Basingstoke, 2008).
7. Jackson, J. C., Watts, J., Henry, T. R., List, J. M., Forkel, R., Mucha, P. J., et al. Emotion semantics show both cultural variation and universal structure. *Science* **366**, 1517-1522 (2019).
