# Supplementary material for: Natural emotion vocabularies as windows on distress and well-being
Source: Nat Commun. 2020 Sep 10;11:4525. doi: 10.1038/s41467-020-18349-0 (PMC7483527; doi:10.1038/s41467-020-18349-0)
Supplement: Supplementary file 3 — Reporting Summary [file 41467_2020_18349_MOESM3_ESM.pdf]

nature research

Corresponding author(s): Vera Vine, Ph.D.

Last updated by author(s): 04/27/2020

Reporting Summary

Nature Research wishes to improve the reproducibility of the work that we publish. This form provides structure for consistency and transparency in reporting. For further information on Nature Research policies, see Authors & Referees and the Editorial Policy Checklist.

Statistics

For all statistical analyses, confirm that the following items are present in the figure legend, table legend, main text, or Methods section.

n/a	Confirmed	
☐	☒	The exact sample size (*n*) for each experimental group/condition, given as a discrete number and unit of measurement
☐	☒	A statement on whether measurements were taken from distinct samples or whether the same sample was measured repeatedly
☐	☒	The statistical test(s) used AND whether they are one- or two-sided<br>*Only common tests should be described solely by name; describe more complex techniques in the Methods section.*
☐	☒	A description of all covariates tested
☐	☒	A description of any assumptions or corrections, such as tests of normality and adjustment for multiple comparisons
☐	☒	A full description of the statistical parameters including central tendency (e.g. means) or other basic estimates (e.g. regression coefficient) AND variation (e.g. standard deviation) or associated estimates of uncertainty (e.g. confidence intervals)
☐	☒	For null hypothesis testing, the test statistic (e.g. *F*, *t*, *r*) with confidence intervals, effect sizes, degrees of freedom and *P* value noted<br>*Give P values as exact values whenever suitable.*
☒	☐	For Bayesian analysis, information on the choice of priors and Markov chain Monte Carlo settings
☒	☐	For hierarchical and complex designs, identification of the appropriate level for tests and full reporting of outcomes
☐	☒	Estimates of effect sizes (e.g. Cohen's *d*, Pearson's *r*), indicating how they were calculated

Our web collection on statistics for biologists contains articles on many of the points above.

Software and code

Policy information about availability of computer code

Data collection	No software was used.
Data analysis	Two softwares were used to process data for analysis. (1) Linguistic Inquiry and Word Count, Version 15, is a widely used, commercially available software program (LIWC; Pennebaker, Booth, Boyd, & Francis, 2015; full citation in References section). (2) Vocabulate is an open-source software program we developed for the purpose of this investigation; it is described in the Methods. Vocabulate may be downloaded free of charge from https://osf.io/8ckyp/ or https://github.com/ryanboyd/Vocabulate. Following Nature Research guidelines, we also submit a software summary form and zip file with the required information for reviewer/editor purposes.

For manuscripts utilizing custom algorithms or software that are central to the research but not yet described in published literature, software must be made available to editors/reviewers. We strongly encourage code deposition in a community repository (e.g. GitHub). See the Nature Research guidelines for submitting code & software for further information.

Data

Policy information about availability of data

All manuscripts must include a data availability statement. This statement should provide the following information, where applicable:

- Accession codes, unique identifiers, or web links for publicly available datasets
- A list of figures that have associated raw data
- A description of any restrictions on data availability

The datasets analyzed during the current studies are available at a persistent community repository via OSF.org at https://osf.io/8ckyp/.

Field-specific reporting

Please select the one below that is the best fit for your research. If you are not sure, read the appropriate sections before making your selection.

☐ Life sciences ☒ Behavioural & social sciences ☐ Ecological, evolutionary & environmental sciences

For a reference copy of the document with all sections, see nature.com/documents/nr-reporting-summary-flat.pdf

Behavioural & social sciences study design

All studies must disclose on these points even when the disclosure is negative.

Study description	This study analyzed quantitative indices derived from open-ended samples of participant language. Study 1 used indices derived from one-ended, stream-of-consciousness essays; Study 2 used derived the same indices from publicly posted Internet blogs. Study 1 text data were used correlationally in conjuction with self-report questionnaires.
Research sample	Both studies analyzed data from existing datasets. Study 1 analyzed data from 1,567 university undergraduates (mean age 18.8, SE = 2.0) who were 60.7% female. Study 2 analyzed data from 35,385 publicly posted blogs; more information on this dataset can be found in Schler, J., Koppel, M., Argamon, S., & Pennebaker, J. W. (2006). Effects of Age and Gender on Blogging. Proceedings of the 2006 AAAI Spring Symposium on Computational Approaches for Analyzing Weblogs, 86, 191–197. https://www.aaai.org/Papers/Symposia/Spring/2006/SS-06-03/SS06-03-039.pdf. Both Study 1 and Study 2 datasets generated during and analyzed during the current studies are available in The Open Science Framework repository, at https://osf.io/8ckyp/.
Sampling strategy	Study 1 used a convenience sampling procedure; participants were students enrolled in a large online undergraduate course. Study 2 used an age- and gender-stratification approach; more information can be found in Schler, J., Koppel, M., Argamon, S., & Pennebaker, J. W. (2006). Effects of Age and Gender on Blogging. Proceedings of the 2006 AAAI Spring Symposium on Computational Approaches for Analyzing Weblogs, 86, 191–197. https://www.aaai.org/Papers/Symposia/Spring/2006/SS-06-03/SS06-03-039.pdf. A priori sample size calculations were not done; however, as described in the manuscript (p. 6), even for the smaller Study 1 sample, sample size was sufficient to detect even small correlations with adequate (.80) power.
Data collection	Open-ended text data were typed by participants into their personal computers into an online survey platform for Study 1, and commercially owned blogging website (Blogger.com) for Study 2. Study 1 additionally used survey responses collected via the online survey platform. Blinding of researchers to data and study hypotheses are not applicable, given that data were collected remotely and archivally prior to the development of the current investigation, and the investigators did not interact with the participants.
Timing	Study 1 data were collected over the course of a single semester, with the Time 1 writing occurring mid-September and Time 2 writing in early December of 2014. Self-reported questionnaires were administered in between those dates. Study 2 blogs were collected on a single day in August, 2004. Blogs varied in the number of entries for each blogger that ranged from a single entry on a single day to thousands of entries over at least 7 years.
Data exclusions	A priori exclusion criteria were consistent with existing practice in computerized text analysis research. Exclusion criteria were: each text much include at least 100 words; at least 70% of which must be identifiable by the default LIWC [i.e., software] lexicon. In Study 1 these criteria led 12 (0.76%) of the original 1579 to be excluded. In addition to the above exclusion criteria, Study 2 also further excluded texts that were duplicates collected in error; the combination of all Study 2 criteria led 1,911 (5.12%) of the original 37,296 blogs to be excluded.
Non-participation	No participants dropped out or declined participation. However, because the data for Study 1 were collected over the course of the semester, some missingness is present in self-report questionnaires and Time 2 essays based on student attendance. Trait differences between Time 2 essay completers and non-completers are reported in the Method section of Study 1.
Randomization	Participants were not allocated into experimental groups.

Reporting for specific materials, systems and methods

We require information from authors about some types of materials, experimental systems and methods used in many studies. Here, indicate whether each material, system or method listed is relevant to your study. If you are not sure if a list item applies to your research, read the appropriate section before selecting a response.

Materials & experimental systems

n/a	Involved in the study	
☒	☐	Antibodies
☒	☐	Eukaryotic cell lines
☒	☐	Palaeontology
☒	☐	Animals and other organisms
☐	☒	Human research participants
☒	☐	Clinical data

Methods

n/a	Involved in the study	
☒	☐	ChIP-seq
☒	☐	Flow cytometry
☒	☐	MRI-based neuroimaging

Human research participants

Policy information about studies involving human research participants

Population characteristics	See above
Recruitment	See Sampling Strategy, above. Regarding the potential for bias or self-selection in recruitment, note that the Study 1 participants, who were students enrolled in an online Introductory Psychology course, reflected the demographics of lower division students at the University of Texas at Austin in terms social class (as measured by parents' education), age, and ethnicity. The percentage of females taking the class, however, was higher than the remainder of their cohort (our study = 60.7%; freshman/sophomore student body = 54%). The vast majority of students who took Introductory Psychology during the semesters the course was offered took the online course because the alternative sections were reserved for an honors program.
Ethics oversight	The study protocol was approved by the Institutional Review Board at the The Institutional Review Board at the University of Texas at Austin.

Note that full information on the approval of the study protocol must also be provided in the manuscript.
